# Entanglements in Health and Well‐being: Working with Model Organisms in Biomedicine and Bioscience

**DOI:** 10.1111/maq.12489

**Published:** 2019-02-27

**Authors:** Carrie Friese, Joanna Latimer

**Affiliations:** ^1^ Sociology Department London School of Economics and Political Science; ^2^ Department of Sociology University of York

**Keywords:** model organisms, laboratory animals, care, interspecies occupational health

## Abstract

Drawing on collaborative ethnographic fieldwork, this article explores how human health becomes entangled with that of model organisms in day‐to‐day biomedical science. Social science scholarship on modeling has explored either how specific models impact and shape our knowledge of human disease or how animal technicians and scientists affect laboratory animals. This article extends this relational approach by asking how embodied and institutional care practices for model organisms affect the health and well‐being of animal technicians and scientists. We focus on two interspecies bodily experiences: pathogenic exchange and stress. We then explore enrichment as a strategy for producing health and well‐being across species. We suggest that relations of care form a crucial part of biomedical knowledge production. Not only does care figure in the shaping of model organisms; care for technicians and scientists also plays a role in bioscientific knowledge production. We conclude by proposing an interspecies approach to occupational health.

[A]ll animal experimentation develops entanglements between human and animal capacities, with potential consequences for both animal and human well‐being—which can never be ignored by science. (Davies 2012a, 633)

## Introduction

Laboratory animals used in biomedical science have enabled human and animal health to be understood as entangled. Specifically, animal models and other model organisms are central to translational medicine, where nonhuman lives of various kinds serve as model systems for understanding biological processes across a range of species (Ankeny 2007; Ankeny and Leonelli 2011; Friese and Clarke 2012). In this work, we learn that biomedicine relies on the nonhuman animal who is born, suffers, and dies for the life and health of human animals (Svendsen and Koch 2013). Social science scholarship on modeling in medical research has explored *either* how specific organisms impact and shape our knowledge of human disease (Ankeny et al. 2014; Davies 2010, 2012b; Nelson 2018) *or* how animal technicians and scientists affect laboratory animals (Dam and Svendsen 2017; Druglitrø 2017; Greenhough and Roe 2011; Kirk 2008, 2010, 2012a, 2012b, 2014, 2016).

This article extends this body of scholarship by exploring how model organisms affect the health and well‐being of animal technicians and scientists. We build on Elena Buch's (2013, 639) argument, developed in the context of her ethnographic research on elderly caregiving, that: “Taking the relational nature of personhood seriously requires expanding discussions regarding the social relations and processes that constitute independent persons to include the ways that these processes might also affect caregivers.” Indeed, as one of us has argued elsewhere, the conditions of care are as critical for the so‐called caregiver as the person who “needs” caregiving (Latimer 2011, 2018). We transpose this argument from elder care to care for model organisms, similarly arguing that social science approaches consider how care practices affect both model organisms *and* animal technicians and scientists.

Drawing on collaborative ethnographic fieldwork, we explore the specific ways in which both model organisms as well as scientists and animal technicians are constituted as (un)healthy in day‐to‐day laboratory practices. We focus here on the relationships between animal technicians and mice in the biological services unit (BSU) or “animal house” of the Institute, as well as the relationships between scientists and worms used as model organisms in a laboratory. Using Svendsen and Koch's (2013) delineation of “corporeal exchange” between pigs and scientists, we thus consider the entanglements between humans and model organisms in research settings. We focus on two shared and interspecies bodily experiences: pathogenic exchange and stress. We then explore enrichment as a strategy for producing health and well‐being across species, not only in the animal house (where the term enrichment is prevalent and readily used) but also in the working lives of technicians and scientists. We conclude by proposing an interspecies approach to occupational health that is based on these findings.

## Model Organisms and Caring for Laboratory Animals

The most obvious entanglement of human and animal health in biomedical research is conceptual. Within the biomedical domain, laboratory animals are constituted as sacrifice‐able species that can stand for and represent human bodies and diseases (Birke et al. 2007, Lynch 1989; Svendsen and Koch 2013). The use of animals as experimental models in biomedical research is justified by this distinction between species (Ingold 2000)—nonhuman animals can be modified, experimented on, and eventually killed in ways that humans can and should not be (Svendsen and Koch 2013).^1^ At the same time, modeling also troubles the idea of a clear species delineation because of a presumed interspecies homology in molecular substance (genomes) and/or process (such as reproduction, development, and aging) that makes modeling relevant (Svendsen and Koch 2013). In this context, the differences between humans, experimental animals (e.g., sentient species like mice, pigs, cats, and dogs) as well as other living experimental organisms assumed to be insentient (e.g., flies, worms, yeast, bacteria) is generally emphasized, if always also open to question through comparison (Ankeny 2007).

Much of the sociological, historical, and philosophical scholarship on animal modeling has focused on the epistemic and organizational aspects of modeling in biomedicine. Model organisms are usually thought of here as “epistemic objects” (Rheinberger 2010, 154), around which entire disciplines can be built and different disciplines can come together (e.g., Kohler 1994; Shostak 2007). A key distinction made by these scholars is that between models representing biological processes occuring in another target species (e.g., humans) versus models that stand for a more basic process occuring across a full range of species (Ankeny and Leonelli 2011; Bolker 2009). The representational credibility of both types of models is constantly in question, however, and risks being dismantled if claims based on one species are over‐generalized to another species (Nelson 2013). Species generality through the evolutionary conservation of biological processes is thus a key assumption in modeling practices (Logan 2001, 2002, 2005), but one that is always also a site of concern, question, and debate (Ankeny 2007; Dam and Svendsen 2017).

Sociologists, historians, and cultural geographers have also probed the entanglement of humans and animals in biomedical research by exploring how laboratory animal (e.g., mice and flies) bodies have been “standardized” to better represent human bodies, particularly in the context of biomedical “translations” (Clause 1993; Dam and Svendsen 2017; Kirk 2010, 2008, 2012b; Logan 1999, 2002; Rader 2004). Selective breeding and transgenics enables animal bodies to be imagined as homogeneous in an effort to stabilise knowledge (Davies 2012b, 2013a, 2013c; Rader 2004). Animal bodies are also standardized through routinized animal care that focuses on uniform practices of housing, food, and handling (Dam and Svendsen 2017; Davies 2010, 2013b; Druglitrø 2017; Kirk 2008, 2010, 2012b, 2014, 2016). However, Gail Davies (2010, 2011, 2012a, 2012b, 2013a, 2013b, 2013c), Nicole Nelson (2018), and Mie Dam and Mette Svendsen (2017) have all shown that standardization is often unobtainable, even when genetics are held constant, as the very different spaces in which humans and laboratory animals live often thwarts translational efforts.

Across the human–animal entanglement scholarship, care has become a central theme (Druglitrø 2017; Friese 2013; Giraud and Hollin 2016; Guerrini 2003; Holmberg 2011; Kirk 2016; Svendsen et al. 2018). *Care for* laboratory animals is generally understood in utilitarian terms within medical science, where concern with the well‐being of animals is crucial for producing high quality science (Friese 2013; Giraud and Hollin 2016; Kirk 2016). How nonhuman animals are cared for in laboratory science is thought to matter for biomedical knowledge production and is thus consequential for “representational targets” (e.g., human patients) (Dam and Svendsen 2017; Davies 2013b).

Adding to this literature, we explore how caregiving affects not only the model organisms, but also the health and well‐being of their caregivers (specifically animal technicians and scientists) in ways that shape biomedical knowledge. In contemporary STS literatures, care is understood as a highly relational, highly productive practice that constitutes the worlds within which we live, but also a practice that is very messy, complicated and uncertain (Haraway 2008; Latimer 2000, 2013; Latimer and Miele 2013; Puig de la Bellacasa 2011, 2015). STS scholarship has argued that care is not innocent and cannot be valorized as a way out of unequal relations; care can just as often also cement hierarchies (Giraud and Hollin 2016; Martin et al. 2015, Murphy 2015). These arguments intersect with longstanding feminist explorations of care work across a range of disciplines, which emphasize the unequal relations that caring practices both rely on and reproduce (Buch 2013; Colen 1995; Gilligan 1982; Hochschild 2012; Vora 2015).

In her review, Elana Buch (2015, 279) argues that care is thus a shifting and unstable anthropological concept, one that can refer to everyday practices, engagements with biomedicine, biopolitics, affective states, forms of moral experience and obligation, structures of exploitation, and the relationships between these various things. Like Buch, we do not insist on one definition of care, but instead explore the multiple ways in which care gets enacted and articulated as affective concern and practical action within our field site (see also Tronto 1994). But where Buch (2013, 2015) sees care as important for generating *social personhood* that is distinctly human, we build on Maria Puig de la Bellacasa's (2017) work to explore care as generating *more than human socialities*, where “neglected things” like mice and particularly those animals assumed to be insentient, such as worms, are involved in constituting not only the human person but also scientific knowledge and organization. We stress how care can appear as “a selective mode of attention,” one that “circumscribes and cherishes some things, lives, or phenomena as its objects” as it excludes others (Martin et al. 2015, 627). We therefore explore care as an emergent property of complex interactions, and do not overdetermine when or how care represents ethical doings (Latimer and Bellacasa 2013). This interpretation of care means that such work variously focuses on the health of the human or nonhuman animal, the model as metonymic for the experiment, or even the individual's, the laboratory's and/or the institution's ambitions and reputation.

## Materials and Methods

This article draws on ethnographic fieldwork from 2015 to 2017 at a life sciences research institute in the United Kingdom, conducted by both Friese and Latimer. We attended several meetings at the Institute, through which we introduced our research and discussed how we would undertake a study of how the Institute modeled aging and cared for the animals used in their research. Over the next year and half, we shadowed animal technicians, laboratory heads, postdoctoral and post‐graduate research scientists, and a veterinarian. As well as positioning ourselves at the benchside in the laboratories, Friese immersed herself in the Institute's biological services facility (BSU), or “animal house,” where mice were bred, modified, and euthanized in preparation for their use at the bench. Friese then “followed” these mice into various laboratories. Latimer also conducted semi‐structured, tape‐recorded, qualitative interviews at the scientists’ offices at the Institute after periods of participant observation in the laboratories. These scientists, observed and intervewed by Latimer, worked with mice, yeast, worms, and cells derived from human blood.

Throughout the research we followed the models to understand how care is practiced in this specific research site. In the context of our research, mice were unique organisms; as regulated species their breeding and care was largely provided by animal technicians and veterinarians in the animal house from which the scientists were inadvertently excluded. In all but one instance, both authors saw only dead mouse body parts when shadowing scientists who used the mice in their experiments. Care of living things did not arise as a generative concept in these laboratory contexts. In contrast, worms, as an unregulated animal species, are sustained as living things by scientists in the laboratories because they are instituted as insentient. We observed how worms are enacted by laboratory processes and practices as not only instrumental objects but also as *living* organisms that require care. Care thus did arise as a generative concept in these laboratories (see also Latimer and Puig de la Bellacasa 2013). As such, care practices were unevenly distributed and very much linked to the type of model organism used by the laboratory in their experiments, which was linked to the regulation and corresponding spatial distribution of different species across the Institute.

Comparing care across species as well as across different kinds of scientific sites, including foregrounding care of worms grown and used in the laboratory, requires that we make certain awkward juxtapositions and comparisons (Strathern 1997) between care of different kinds of living things: human and nonhuman animal but also human technician, veterinarian and scientist; regulated, sentient, vertebrate animals like mice versus unregulated, insentient, invertebrate animals like worms; occupational health and animal welfare; care for others, and care for self. In comparing and juxtaposing human and nonhuman animals, we are deliberately troubling the very divisions that underpin and dominate social as well as biological taxonomies.

## Pathogenic Exchange

The risk of pathogenic exchange between animals and humans is high among those caring for animal models in laboratory research. At the same time, risk can be mediated through care. Controlling pathogenic exchange is indeed central to the work of the Institute's animal house. Not only does the facility exclusively use ventilated cages and racks that provide filtered air and water to the mice, but it also has a whole set of procedures in place meant to minimize pathogenic exchanges between the laboratory animals and their human caregivers. In this section, we will explore how the institute has responded to high levels of animal allergies among BSU staff, which is caused by interaction with the mice. We show that controlling pathogenic exchange is directed at *both* the human and the animal in modeling practices, with the aim of facilitating health among interacting species.

Robert Kirk has shown how, since the middle of the 20th century, limiting and controlling the intercorporeal mixing of animals in laboratories has been central to standardizing animals as model organisms. This has been done through the creation of germ‐free animals as well as the conversion of isolation technologies into laboratory animal cages (Kirk 2012a, 2012b). Isolating laboratory animals from specific pathogens has also been crucial in maintaining genetically modified animals, several of which are immunocompromised (Davies 2012b). Controlling pathogenic exchange has thus been understood as a key way of acting on laboratory animals, transforming them into standardized models for biomedical research.

Kirk (2016) as well as Giraud and Hollin (2016) have shown that healthy animals and quality care have been equated in laboratory animal welfare since the mid‐20th century. This was seen at the Institute, which attributed the health of its animals to its biosecurity measures, which they and other scientists understood as high quality care. Indeed, it was recommended that we conduct participant observation at this site in part because the longevity of the mice at this facility was far greater than many would expect, and the Institute was thus deemed exemplary in providing animal care.

Friese has to learn this form of care to enter the mouse house, which involves passing correctly through the space of the locker room as well as the air shower. Escorted by Evelyn, an animal technician, Friese is told that she can enter the BSU through the air shower with an employee, but that on leaving she will have to leave through the air shower on her own. Evelyn presses the button for the shower to begin, and air was blown on the two of us from multiple directions for about 60 seconds. As they enter the experimental unit of the BSU, Friese asked why they can enter the BSU through the air shower together, but have to leave the unit through the air shower alone. Evelyn responds that they want to make sure any animal pathogens do not get out with us. Where Friese presumed the biosecurity measures were for the health of the mice, she learns that air showers were also there for the health of the humans.

We generally think that isolating laboratory animals is for the purpose of animal health and standardization. By being more healthy, these animals will be better models—capable of improving the health of human patients *downstream*. But the air shower shows how controlling pathogenic exchange is also for the purpose of creating healthy caretakers. Indeed, before undertaking fieldwork in the animal house, Friese has to have a number of tests taken at occupational health and safety: blood tests, a lung test, and an hour‐long mask fitting test along with training on how to properly fit a mask. She is told that occupational health has been slow to address the working conditions of animal technicians and that this has posed problems for the Institute; a rising number of people in the BSU have developed severe animal allergies. Bruce, the head of occupational health, tells Friese that some of the most senior BSU staff can barely enter the animal house anymore, as they have developed such severe animal allergies. Both the laboratory animal and the animal technician need to be cared for by the institution in this context, by carefully modulating interspecies relationships for the purpose of interspecies health. This is required for both species (animal technician and animal model) to care for another (animal model and downstream human patient).

The effort to control pathogenic exchange between humans and animals, for the health of both species, has the unintended consequence of keeping many of the scientists out of the BSU at the Institute. The shower was a continual point of reference in our discussions with scientists, who frequently told us both formally in the interviews conducted by Latimer and informally during participant observations that they would like to see their mice a lot more. However, as one scientist put it, they simply do not have time to “take all those showers.”

The problems of physical contact in the context of biosecurity has tended to focus on the ways that infant–maternal attachments—particularly in laboratory primates—are interrupted through attachment theory (Kirk 2012a), and hence on the consequences of limited relationships *within* species for the psychological well‐being of laboratory animals. We find that delimited physical contact *across* species also shapes day‐to‐day experiences in producing bioscientific knowledge, isolating scientists from the animals that their research is based upon. The scientists are thus unable to engage in the kind of caring relations with their mice that at least some would like. This could be seen as a site of unintended *dis*‐entanglement.

## Stress

Stress is another issue where the health of human scientists, animal technicians, and laboratory animals is entangled. Kirk (2014) has shown how the idea of stress transformed the ethics and eventual regulation of laboratory animal use in British science, expanding the scope of how an animal may suffer beyond physical pain—including social and mental distress. Building on ethological knowledge, stress in these studies included not only how the animals related to one another in the cage but also how the animals related to the scientists and animal technicians working with them. From the 1960s to the 1980s, the focus shifted from not only mitigating animal pain to also promoting animal *well‐being* (Kirk 2014, 251). Kirk focuses on how the human is imbricated in the production of animal well‐being in this context:

Stress made the physical and social environment determining factors of the physiological state of the laboratory animal under study. Furthermore, stress relocated the human subject within that environment, making the researcher integral to, controller of, and obligated to, the laboratory animals’ well‐being. (Kirk, 258, see also Nelson 2018, 115–16 especially)

We want to emphasize here that model organisms also create stressors for animal technicians and scientists in ways that shape the latters’ health and well‐being.

### Stress in the BSU

Friese is spending the day with an animal technician, Janet, who has done this type of work for about 35 years. Janet starts by giving some background to her work for the day. The Balb C mice that she looks after in the animal house are destined for use in one of the laboratories in the Institute, which focuses on questions of aging and immunity. Janet explains that the problem with Balb C mice is that males of this strain cannot be aged because they fight too much, even with litter mates, so aged male Balb C mice would have to be housed alone. Housing mice for up to three years alone in a cage was not something that the Institute considered ethical, the implication being that mice need community or they will become excessively stressed. So the lab, in conjunction with the BSU, decided to try to age female Balb C mice, who are considered quite docile, very unlikely to fight, and thus can be housed together. This was the first time that females of this strain had been aged by the animal house, and Janet says that it has been a big learning process for them.

For example, once they started aging Balb C mice, the technicians began finding bloody discharge in the bedding. The technicians became very concerned and called the veterinarians in. By performing autopsies on these mice after they died, the veterinarians realized that they had developed ovarian and liver cancers in their old age. Since the veterinarians did not believe that the mice were experiencing any pain or suffering from the tumours, the research could continue. Nevertheless, the technicians began to check the cages every day to ensure the health of the mice. Balb C mice are fragile, however, and this level of surveillance proved to be excessive: too *stressful* for the animals. Modifying their technique of care, the technicians now check the cages one time per week.

Janet starts these weekly checks by organizing the cages according to age, starting with the oldest ones first. All of the cages with older mice are marked with a red tag stating, “ageing illness, bloody discharge.” Janet puts eight cages on the trolley, and brings the mice close to the hood. She turns off the light on the hood so as not to disturb the quite anxious Balb C. Janet further explains this precaution, saying, “because they're albino, it just feels like they should not have light directly on them.” She puts one cage under the hood, and looks at the mice in the cage, watching their gait and how they are moving. One at a time, she scruffs each mouse to look at her underside, palpates her stomach to check for any hardness, and then looks at her teeth. Janet watches the mouse move around the cage, and holds the mouse in her hand to see how she comes off to assess strength. If Janet notices anything wrong with a mouse, she takes note and contacts her line manager to decide on the required next steps. In one instance, Friese sees Janet and her line manager decide to call on one of the two full‐time veterinarians, who will determine whether or not a mouse is suffering to the point where it should be killed in accord with U.K. regulations.

Janet is *affected* by the mice's predicament. Friese asks Janet what happens if the technicians and veterinarian feel an aged Balb C mouse simply will not make it to be two years old, and thereby will not able to be used in the aging research. Janet replies that there is one mouse that she thinks will die before two years, and she adds that there are times when the lab is told by the BSU that they will just have to do something different because the mice will not live to two years. The lab will try to do whatever it can with the tissue from those mice. Janet continues that it is hard to see a mouse who is starting to die, and who won't quite make it to an experiment. “To have lived in these cages for two or more years, which must be *boring* for the mice, and then to not quite make it to the study, it is really sad.”

Stress is being enacted in multiple ways within these interspecies relations. The physical body of the mouse is stressed by age and the emotional body of the mouse is stressed by the boredom of living, for a very long time, in a small cage. Both these stressors affected Janet, which, in turn, creates an everday form of work‐place stress. For Friese, this stress appears palpable, as Janet talks throughout the day about the research she is doing, in her own time, to provide more interesting and stimulating treats for “her mice” to help alleviate their boredom. Like Claire Stacey's (2011) and Elena Buch's (2013) findings with human care givers, we find that animal technicians “contribute to their own exploitation” by taking on additional and unpaid work to attend to their affective ties with their mice.

While much of animal technicians’ care work is physical labor—it is physically hard to be on one's feet, to bend up and down repeatedly to get and lift cages, to fit one's body within the hood all day—animal technicians’ work also entails emotional labor. It is emotionally hard to work with stressed animals—mice whose strain is prone to be anxious, who are in a small cage for as long as a mouse can be made to live, and who have cancers as a result. And this creates stress for the animal technicians. The animal technician is the “controller” (Kirk 2014) of the laboratory animal's well‐being, but this is a form of entanglement that also causes emotional, physical, and psychic stress for the caregivers. Stress and well‐being are here relationally produced across species.

### Stress at the Bench

Doing bench science is perceived by many scientists as stressful. In all the interviews conducted by Latimer—with scientists at different moments in their scientific careers—the stressful demands of being a scientist were articulated as something that needed to be managed. Stressors were rather expectedly connected to funding and publishing cycles and the problem of failure, particularly failed experiments. But what emerged in our data, and what we focus on here, is how the life and disease cycles of model organisms and the demands of an experiment make specific kinds of demands on scientists.

Jake is a Ph.D. student and is in the process of becoming a scientist. When shadowing Jake, he appeared incredibly confident and adept. On one occasion, Latimer was observing him work with gravid worms, with the aim of making a cohort of aged worms from his RNA libraries. The worms come from a particular strain bred to be sterile at 25 degrees centigrade. Jake gets a plate of worms from the incubator, which keeps the worms at a steady temperature, and takes them to the bench beside his microscope. He notes that he is pleased because the worms have reproduced well in the incubaror and on the food he has given them; his care of the worms has worked.

Jake tells Latimer that he is going to dissolve the worms in sodium hypochlorite, or bleach, leaving just their eggs. He continues to explain that this is not very nice, bleaching worms to death, but he needs a population of eggs that, when they hatch, will be in a different temperature than normal—a temperature of 25° Celsius—so that they become sterile. Jake will do this for different batches of worms at different moments in their life cycle. This will allow him to observe differences in gene expression that are not attributable to the young tissue/embryonic tissue contained in the eggs. He says that it is simple to do this in the worm. All tissue is somatic except for the germline tissue (the tissue in the eggs); somatic tissue does not divide, so one set of cells lasts the worm's lifetime. Thus, the demands of Jake's experiment fit the specificites of the worm's biology (life cycle, reproductive characteristics), which requires an exacting control of the environment (supplies of food, temperature, etc). That is, it requires *matters of care* for both the worms and the experiment that are so sedimented and normalized that these care practices have becomeinvisible.

Later, during the recorded interview, Jake sums up what he calls the “doing of science.” Importantly, this interview took place after Jake had discovered that the experiment, partially described above, had failed. This meant that half of his work for his Ph.D. had failed. Jake was very, very low; his body language and tone were deflated. At this moment, he describes in the interview the doing of science as “being married to your model.”

Jake: If I'd been told what it takes to do science. Not to be a scientist, but to do the science. … Like the time and the frustation, and the … especially working with biology. I think I would have considered it a lot more heavily. I may have made the same decision, but … I don't think I ever. … That was never really impressed upon me as a student, was the doing of the science. Because in school all you do is learn, it's book work, and it's what I loved! [Laughs].

[Latimer]: Yeah, so you mean the kind of, the real practical …

Jake: Yeah.

[Latimer]: Working the materials, working between all these different kinds of, I mean it is extraordinary, you know, in a day, following one of you.

Jake: Mmm.

[Latimer]: How you're working with so many different techniques. Different machines, different procedures.

Jake: Yeah, well not just that, I think the life of a scientist, and like probably, even *just the fact you will be married to your model*.

[Latimer]: Yeah, married to your model!

Jake: It's not something you understand until you do it. But *it dictates your whole life*. I don't like that.

[Latimer]: This is the *Scientific Life*?

Jake: Yes. For people to understand, like it's, this is, it's kind of, doing science is really, sometimes really tough and frustrating, and not nice. … And I don't know, I don't know if that means. … Everyone feels differently about it, and many people have no problem, or at least if they, it's not enough of a problem to sort of not do it. … But when you work with models or you're doing experiments that means you have to come in at the weekend, and almost every weekend, and that sort of just means like it's no longer, you know, *you're not in control of your lifestyle anymore, of your life*, like. And if you don't do the experiments, or you delay them, then they don't get done….The time, the time thing, where there's never enough time, and I don't know if this comes from the funding or just being a student, but yeah, there's always an expectation of, that you'll work more and produce more. That's sometimes quite hard to manage.

We note Jake's metaphor: For him, the image of marriage is the one he reaches for to express the extent to which biology and experimental work entangles him and curbs his freedom. Doing science for Jake at this moment is all about not having control over his own life, about being at the beck and call of the experiment and more specifically the biology of his model organism, the worm. At this moment, Jake's experience is not that of a sovereign subject, with a disembodied, transcendent, conquering gaze from nowhere (Haraway 1991). Rather, for Jake, in the context of a failed experiment, the doing of science that is normally made invisible in publications comes to the foreground, as matters of care that “organizes, classifies, and disciplines bodies” (Martin et al. 2015, 627). These bodies include not only the animal's, but Jake's as well.

While Janet is affected by the boredom of the aged mice in the BSU as an animal technician, Jake is affected by the deterministic role that worms play in his life as a scientist. Stress is both a trope and an experience that partially connects worms, mice, scientists, and animal technicians. The relations that these different species have to one another—scientists with worms, animal technicians with mice—within the confines of the experiment produces embodied states that are defined as “stressful.”

## Enrichment

Enrichment activities are one of of the primary ways in which stress is alleviated among laboratory animals, who are thought to be particularly susceptible to boredom (Wolfer et al. 2004). For example, Lesley Sharp (Forthcoming) notes that televisions are used with nearly all captive primates today to prevent distress and psychological illness caused by boredom in the captive setting. Improving the well‐being of laboratory animals is heavily focused on, ameliorating the boredom of captivity, which is understood as stressful—as exemplified in Janet's example, above. This is why enrichment is a crucial part of laboratory animal welfare and science today. At the Institute, social housing (i.e., not allowing male Balb C mice to live alone for an extended lifetime as described above), cage design, and the inclusion of seeds in cages (that must be searched for and opened, giving the mice something to do) were all examples of how the Institute tries to enrich the lives of its mice.

Enrichment went beyond the mice, however, often targeting animal technicians and scientists as well. The Institute is a campus, with buildings and a residential village arranged in what was the parkland (where sheep graze) of a former aristocratic, mansion house. The lives, health, and well‐being of scientists is enacted over and over again as constitutive of the Institute's identity; indeed, the focus on promoting scientists’ well‐being at the Institute is part of its explicit recruitment strategy, as seen on its webpage. For example, benches and tables are arranged outside in the extensive and well‐maintained gardens that adjoin the parkland, which employees use to sit and think, or work, eat, and socialize. The building in which we did our observations in the laboratories was designed to promote openness, community, and interaction—with offices situated on the other side of the corridors from the labs—either open plan, or with glass doors to promote visibility and easy interaction between all levels of staff. There was a gym, a new running/cycling track, a village with different forms of accommodation to cater to all (from visiting researchers through to married couples with children), an on‐site nursery, a new open plan and modern restaurant purveying healthy options, and sofas and comfortable seating areas for people to meet.

Similarly, the BSU was created to not only provide good care for the animals, but also good care for the animal technicians. Because of the showers, animal technicians could not as easily get out to enjoy the campus life of the Institute. So the facility was built to bring the outside parkland into the BSU as much as possible. This facility was not built below ground, as many animal houses are, but above ground with windows to the outside. There is a nice view to the surrounding parkland at the end of each corridor, as well as from the kitchen where staff take breaks, with tea, coffee, and a television provided. The bucolic image of sheep grazing can thus be enjoyed during a break. Artificial plants, which required special permission from the Home Office that licenses animal houses and all scientists working with animals, are present and meant to make a more pleasant work space for technicians.

The scientists’ and the animal technicians’ working lives were thus enriched, as much as the working lives of the mice were. And for both species, these enrichment activities were for the purpose of enhancing health and well‐being including ameliorating stress. Ameliorating stress, in turn, improves productivity across species. The laboratory animal serves as a better model organism if it is well, in that the biological consequences of stress do not create confounding variables in the research (see also Friese 2013). The animals will be better cared for if there is not a high turnover of animal technicians; keeping animal technicians happy at their job improves the well‐being of the mice. The scientists are similarly more productive if they are less stressed; the kind of stress described by Jake is simply not sustainable across a career.

For the scientists in particular, this included cultivating a “care of self” that was facilitated by the Institute. Latimer is spending the day with Helena, a postdoc. She starts her day by sitting at her desk, taking time to clearly and quietly think through her day. She tells Latimer that there are four things that they need to do, plus two seminars to attend. Latimer responds that Helena really packs it in. Helena says that she likes to get a lot done, to feel good and that otherwise she will feel frustrated.

Throughout their time together Helena touches her belly, a gesture common to pregnant women. She talks about the things she is doing, and what the Institute is helping her to do, to ensure her baby's environment is secure and to promote *its* health, well‐being, and optimal growth. Helena tells Latimer that she trained as a yoga teacher when she was pregnant last time, and now takes yoga classes at the Institute. In addition, she tells Latimer about a mindfulness course put on by the Institute. A little message pops up on her computer screen—from Tiny Pause—reminding her to take a tiny pause throughout her work day. She tells Latimer that Tiny Pause put on a course at the Institute, which included instructions to take time for a pause that enables deep thought. Helena says that they advised the scientists to turn off their mobile phones and not look at their emails except at specified times during the day; they should switch off notifications, except from the Tiny Pause Program itself, which periodically notifies them to take a deep breath, or a tiny pause.

Both Yoga and the mindfulness course—the first initiated by Helena and adopted by the Institute, and the second put on by the Institute and adopted by Helena—are disciplinary forms that promote a care of self that aims to ameliorate stress. Helena says they help her “think well and keep calm and healthy.” As they go through the day, Latimer sees Helena serene yet active, shifting between different forms of care made possible by the ethos of the Institute: washing her hands, putting on two sets of gloves, taking things slowly and methodically when she needs to think, speeding up when she is working in the lab, participating in the openness of community. Helena continuously shifts between and seems to embody community, care of self, care of others, and, of course, care of her experiments.

## Discussion

In this article, we have explored how the health and well‐being of different species are entangled in the laboratory, when model organisms are used to know and improve human health. The social science literature on laboratory animals is increasingly using the trope of entanglement to understand human–animal relations in this biomedical context. Gail Davies (2012a) uses entanglement to produce and render speak‐able a conjecture in which mice not only model for human patients, but where human patients could also model for laboratory mice (see also Greenhough and Roe 2011). Mette Svendsen and Lene Koch (2013) use the trope of entanglement to describe the unrestricted and dynamic relationships between humans and animals in the laboratory that remain barely noticed, which they contrast with the “calculative exchange” of the animal model paradigm. Entanglement has thus allowed social scientists to understand human–animal relationships in the laboratory as dynamic and constitutive, and thus trouble the delineation of species difference. In the process, both Davies (2012a) and Svendsen and Koch (2013) highlight the role of affect in the entanglements of human and animal health.

Drawing on Davies (2012a) and Svendsen and Koch (2013), we have understood entanglement as a process that links humans and nonhumans in material ways and that has affective dimensions. The need to carefully control pathogenic exchanges between humans and mice shows the material entanglements of laboratory animals and their caregivers. But both model organisms and humans nonetheless continue to affect the health and well‐being of one another: A young scientist expresses the stress of “being married to the model”; the technician tells how affected she is by the stress and sickness of the mice she looks after; Balb C mice become stressed when handled too frequently; aged mice get cancer; worms are bleached to death.

In the process, we expanded on what counts as health and well‐being when nonhuman species serve as models in biomedicine. Health has almost exclusively been considered in terms of the animal (as model) and the human patient (as target), with the health of unregulated species like flies and worms being deemed inconsequential. This article has sought to show how animal technicians and scientists alike are affected by their entanglement with other species, and how the health and well‐being consequences of these interspecies relations is experienced by individuals as well as managed by the Institute. In this context, enrichment is not something that humans simply do to laboratory animals to improve and control the health and well‐being of their models. Rather, technicians and scientists are also affected by their models and—as in the case of Jake—sometimes feel that their lives are actually being controlled by the model organism. The technician's and scientist's environment also needs to be enriched in this context, to make the stress of doing science with other species manageable.

This adds another way in which we can understand care as a crucial part of medical knowledge production. Not only does care constitute knowledge by shaping the body of the model organism (Davies 2012a; Friese 2013). Care for technicians and scientists is also constitutive of how scientific knowledge can be produced. Showers delimit the proximity of the relationship between mice and scientists at the Institute, delimiting the possibility for caring relations between scientists and their mice. Care for self through yoga and mindfulness becomes part of scientific practice. Care entangles humans and other model organsisms in the animal house and in the laboratories, as a world‐making activity (Puig de la Bellacasa 2011), which constitutes the health and well‐being of all species involved, and in ways that exceed the model organism paradigm. We thereby extend Elena Buch's argument that care affects the caregiver from the elder care setting to the laboratory animal care setting, and thus argue that caregiving not only makes persons but also makes knowledge.

## Conclusion

By way of conclusion, we would like to suggest the productivity of developing a more than human approach to occupational health. In making this argument, we build on and extend the ways in which Beth Greenhough and Emma Roe (2011) have proposed a “cross‐species research ethics.” Greenhough and Roe here put the ethics of laboratory animal and human subject research side by side, in an awkard comparison, to note that the ethics of human research subjects tends to focus on consent and language, where the ethics of laboratory animal research tends to focus on affective relations. Greenhough and Roe argue that, rather ironically, it could benefit human research subjects to incorporate the focus on affective relations that is seen with sentient laboratory animals.

A cross‐species occupational health approach would similarly put the model organisms working *in* experiments side by side with the technicians and scientists who *are working* the experiments. Occupational health tends to focus on delimiting the conditions through which human workers could become ill in the workplace, which is understood as a political economic issue (Murphy 2006). This is done by protecting bodies (e.g., through the air shower to prevent pathogenic exchange) from illness‐causing agents. Animal welfare similarly delimits the conditions through which laboratory animals become ill, but the absence of illness is not deemed sufficient. Laboratory animals are expected to be “happy” (Poole 1997), or at least happy enough. Rather ironically, it could benefit human workers to incorporate the focus on enriched working environments that is seen with sentient laboratory animals.

Avoiding illness and promoting health may indeed need to become part of occupational health as burnout is increasingly becoming a key occupational health hazzard in the context of neoliberalism. Interestingly, to date burnout has largerly been addressed through the empowerment paradigm (e.g., yoga and mindfulness) that focuses on individual‐level changes and thus depoliticizes work‐related stress (Funahashi 2013, 2). Daena Aki Funahashi notes that it is the individual who is deemed in need of reworking in this context, as opposed to the neoliberal structuring of work and precarity. Rather ironically, a cross‐species occupational health could benefit human workers again by incorporating a more relational understanding of stress. This could emphasize the spatial and structural underpinnings of work‐related stress, and its potential amelioration, for humans and animals alike.

## Note

## Footnotes

1 There is a history of medical science experimenting on humans in ways that have relied on and reproduced structures of inequality based on race, class, sex, and nation (Briggs 2002; Washington 2006). This is often through the equation of ‘othered’ human groups with animals (Ritvo 1987; Rothfels 2008).
